# Exploring Vitreous Haze as a Potential Biomarker for Accelerated Glymphatic Outflow and Neurodegeneration in Multiple Sclerosis: A Cross-Sectional Study

**DOI:** 10.3390/brainsci14010036

**Published:** 2023-12-30

**Authors:** Sezgi Kaçar, Danko Coric, Giovanni Ometto, Giovanni Montesano, Alastair K. Denniston, Pearse A. Keane, Bernard M. J. Uitdehaag, David P. Crabb, Menno M. Schoonheim, Axel Petzold, Eva M. M. Strijbis

**Affiliations:** 1 MS Center Amsterdam, Neurology, Vrije Universiteit Amsterdam, Amsterdam Neuroscience, VU University Medical Center, Amsterdam UMC Location VUmc, De Boelelaan 1117, 1081 HV Amsterdam, The Netherlands; d.coric@amsterdamumc.nl (D.C.); bmj.uitdehaag@amsterdamumc.nl (B.M.J.U.); a.petzold@ucl.ac.uk (A.P.); e.strijbis@amsterdamumc.nl (E.M.M.S.); 2 Dutch Expertise Center for Neuro-Ophthalmology, VU University Medical Center, 1081 HV Amsterdam, The Netherlands; 3 Department of Optometry and Visual Sciences, City, University of London, London WC1E 7HU, UK; giovanni.ometto@city.ac.uk (G.O.); giovmontesano@gmail.com (G.M.); david.crabb.1@city.ac.uk (D.P.C.); 4 Academic Unit of Ophthalmology, Institute of Inflammation and Ageing, University of Birmingham, Birmingham B15 2TT, UK; a.denniston@bham.ac.uk; 5 Department of Ophthalmology, University Hospitals Birmingham NHS Foundation Trust, Birmingham B15 2TT, UK; 6 NIHR Biomedical Research Centre, Moorfields Eye Hospital and UCL Institute of Ophthalmology, London EC1V 9LF, UK; p.keane@ucl.ac.uk; 7 Department of Anatomy and Neurosciences, Neuroscience Campus Amsterdam, VU University Medical Center, 1081 HV Amsterdam, The Netherlands; m.schoonheim@amsterdamumc.nl; 8 Department of Neurology and Ophthalmology, Moorfields Eye Hospital, City Road, London EC1V 9LF, UK; 9 The National Hospital for Neurology and Neurosurgery, University College London, London WC1E 7HU, UK

**Keywords:** glymphatic system, multiple sclerosis, neurodegeneration, optical coherence tomography, vitreous haze

## Abstract

Background: The glymphatic system removes neurodegenerative debris. The ocular glymphatic outflow is from the eye to the proximal optic nerve. In multiple sclerosis (MS), atrophy of the optic nerve increases the glymphatic outflow space. Here, we tested whether vitreous haze (VH) can provide novel insights into the relationship between neurodegeneration and the ocular glymphatic system in MS. Methods: This cross-sectional study comprised 315 persons with MS and 87 healthy controls (HCs). VH was quantified from optical coherence tomography (OCT) volume scans. Neurodegeneration was determined on three-dimensional T1 (3DT1) MRI, lesion detection on fluid-attenuated inversion (FLAIR), and layer thickness on OCT. Generalized estimating equations, corrected for age, were used to analyze associations between VH and metrics for neurodegeneration, demographics, and clinical scales. Group differences were determined between mild, moderate, and severe disability. Results: On the group level, VH scores were comparable between MS and control (*p* = 0.629). In MS, VH scores declined with disease duration (β = −0.009, *p* = 0.004) and age (β = −0.007, *p* = 0.001). There was no relation between VH scores and higher age in HCs. In MS patients, VH was related to normalized gray (NGMV, β = 0.001, *p* = 0.011) and white matter volume (NWMV, β = 0.001, *p* = 0.003), macular ganglion cell–inner plexiform layer thickness (mGCIPL, β = 0.006, *p* < 0.001), and peripapillary retinal nerve fiber layer thickness (pRNFL, β = 0.004, *p* = 0.008). VH was significantly lower in severe compared to mild disability (mean difference −28.86%, *p* = 0.058). Conclusions: There is a correlation between VH on OCT and disease duration, more severe disability and lower brain volumes in MS. Biologically, these relationships suggest accelerated glymphatic clearance with disease-related atrophy.

## 1. Introduction

Multiple sclerosis (MS) is a chronic inflammatory demyelinating disease that affects the central nervous system (CNS) [1,2,3]. MS is most commonly diagnosed in females and at a relatively young age: between 20 and 40 years [4,5]. The pathological hallmarks of MS are white matter (WM) and gray matter (GM) lesions throughout the brain, spinal cord, and optic nerves. Lesions are characterized by varying degrees of focal and compartmentalized inflammatory demyelination and subsequent remyelination or gliosis [6,7,8]. Besides inflammation, there are also neurodegenerative processes that act at least partially independently of focal inflammation [6,7,8].

Neurodegeneration in MS is accompanied by the breakdown of axonal and neuronal proteins, leading to marked neuroaxonal loss [9]. Clearance of these waste products from the brain does not occur through normal lymphatics, since the brain lacks a conventional lymphatic system [10]. The discovery of the glymphatic system, a perivascular route of hydrostatic fluid transport, has shed new light on how waste products are eliminated from the brain. In essence, the glymphatic model has challenged a key concept in cerebrospinal fluid (CSF) research by proposing that hydrostatic pressure has to be considered as a partner along the hitherto dominant concept of osmotic pressure for the transport of compounds [11]. The glymphatic theory describes how cardiac pulse-driven hydrostatic pressure drives the perivascular water through the brain parenchyma and how this water exchanges with interstitial fluid (ISF), picking up metabolites before traveling to the perivenous side [12]. This is facilitated by aquaporin-4 channels expressed on the astrocytic endfeet surrounding the brain vasculature [13]. Impairment of the glymphatic system can induce the accumulation of waste products in brain tissue. Decreased debris clearance has been shown to permit toxic properties to contribute to drive neurodegeneration [14]. There is evidence that the glymphatic system is impaired in MS patients [15]. This is linked to the accumulation of neuroinflammatory and neurotoxic factors and subsequent increases in demyelination and neuronal loss [16]. Impaired glymphatic clearance has been previously associated with higher white matter (WM) lesion volume, gray matter (GM) atrophy, and more severe clinical disability in MS patients [15].

The existence of a retinal glymphatic system had been anticipated when this study was conceived [17,18,19] and has now been confirmed experimentally in rodents [20]. In the retina, debris in the vitreous can cause a subtle degree of clouding (vitreous haze, VH), which is not visible by clinical fundoscopy, but can be detected and measured by optical coherence tomography (OCT) [21]. We have previously demonstrated that this technique can quantify VH in uveitis [22]. Here, we hypothesize that VH may also be related to neurodegenerative debris. Our previous work found no association in MS between VH and a number of markers of CNS inflammation (e.g., between MS patients and controls, optic neuritis patients, or concerning non-ocular CNS inflammation) [23], we now tested whether there was any association between VH and markers of neurodegeneration. If so, the novel discovery of an ocular glymphatic may permit the proposal of a biologically plausible mechanism for the clearance of neurodegeneration waste products.

## 2. Methods

### 2.1. Participants and Assessment

MS patients and healthy controls (HCs) were recruited from the Amsterdam MS Cohort [24]. The Amsterdam MS Cohort is a heterogeneous cohort that comprises MS patients who have been under observation since 2001 and who are longitudinally monitored with multimodal imaging techniques over multiple follow-up visits. Patients with a clinically isolated syndrome suggestive of MS who were aged 18 years and older were eligible for inclusion in this study. Exclusion criteria were a relapse or corticosteroid treatment in the month prior to inclusion. Other exclusion criteria included pregnancy, any other neurological or neuropsychiatric disorder (e.g., neuromyelitis optica spectrum disorder, severe depression, or anxiety), a history of alcohol or drug abuse, and CNS comorbidity (e.g., vascular or traumatic abnormalities) on MRI that could not be attributed to MS. High refractive errors (<−6.0 or >+6.0 dpt) and ocular pathology affecting the vitreous or retina were also exclusion criteria. In addition, HCs were not allowed to be related to a patient with MS in the first or second degree of consanguinity. Over the years of follow-up, patients have undergone up to 5 follow-up visits that included clinical assessments, questionnaires, neuropsychological evaluations, and imaging such as MRI and OCT.

For this specific cross-sectional study, we used the available clinical, OCT, and MRI data from the first visit where a specific subject had also undergone an OCT scan. These visits took place between 2011 and 2017.

Assessment of physical disability in MS patients was done on the day of imaging using the Extended Disability Status Scale (EDSS) [25]. Disease duration was defined as the time from first symptoms until the date of visit. The history of MS-associated optic neuritis (MSON) was established according to a consensus protocol [26]. 

This study was approved by the medical ethics committee (protocol number 2010/336) and the scientific research committee (protocol number CWO/10-25D) of the VU University Medical Center. All participants gave written informed consent before entering the study.

### 2.2. Retinal Layer Thickness and Vitreous Haze Measurements

All OCT scans were obtained with a Spectralis spectral domain OCT device (Heidelberg Engineering, Heidelberg, Germany; acquisition software version 1.7.1.0), with dual-beam simultaneous imaging and the eye-tracking function enabled for optimal measurement accuracy [27,28]. 

Peripapillary retinal nerve fiber layer (pRFNL) thickness was measured on a circular, 12° scan (1 B-scan, 1536 A-scans, no predetermined automatic real time) centered around the optic nerve head. The combined macular ganglion cell–inner plexiform layer (mGCIPL) and macular inner nuclear layer (mINL) thickness was derived from a 20 × 20° volume scan (512 A-scans, 49 B-scans, vertical alignment, automatic real time 16) centered around the fovea. Retinal layer segmentation was performed by software provided by the manufacturer (Heidelberg Retinal Angiograph/Spectralis Viewing Module version 6.9.5.0.) After segmentation, quality control was performed according to consensus criteria [29]. mGCIPL and mINL thicknesses were calculated by averaging the thickness for all 8 sectors of the 1.0 mm, 2.22 mm, and 3.4 mm grid, excluding the central 1.0 mm circle.

**Figure 1:**
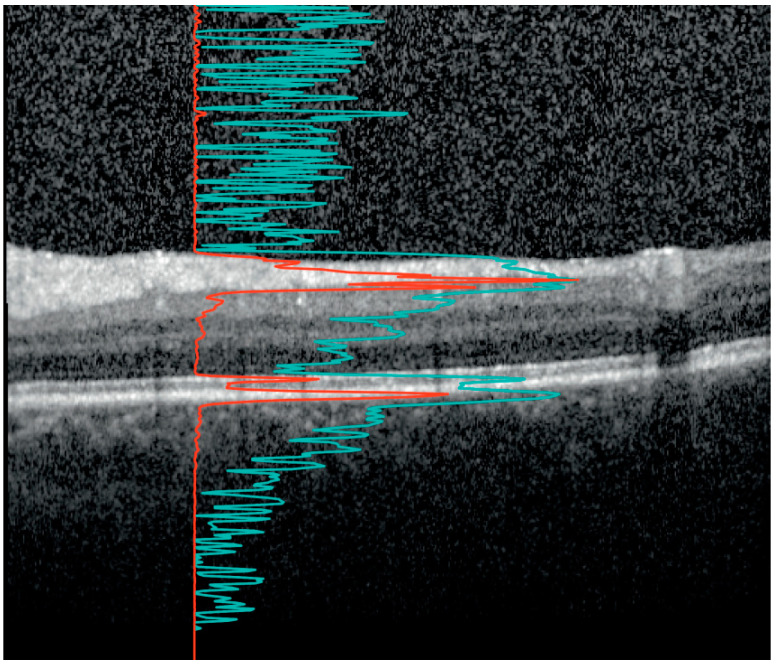
The vitreous haze (VH) signal on optical coherence tomography (OCT). An OCT B-scan between the macula and optic disc. The image is optimized by the device developer for viewing on the computer screen such that the retinal layers are clearly visible. The red line shows the A-scan intensity profile in RAW format of the underlying vertical column in the image. Notice how the sharp peaks correspond to the inner segment/outer segment junction and retinal pigment epithelium at Bruch’s membrane complex. The signal from the red line was used to calculate the ratio of the signal intensity inside the vitreous (above the internal limiting membrane (ILM)) to the mean values of the area below. The light blue line, with sharper peaks in the vitreous and below the retina, represents the same profile after contrast adjustment for visualization. Notice how the vitreous signal is artificially increased compared to the retinal signal. In our study, the VH is calculated based on the raw OCT data (red line).

The VH measurements were also derived from the macular volume scans. Figure 1 illustrates how the VH scores were calculated. The scores were calculated from the raw images recorded by the Spectralis rather than from the contrast-corrected, standard OCT images, as described in Keane et al. [30]. Raw images were exported from the Heyex software (Heidelberg Engineering, Heidelberg, Germany) as VOL files. The measurement used in this work was obtained as the ratio of the mean intensity of all pixels of the vitreous (i.e., pixels above the segmented internal limiting membrane (ILM)) to the mean values below the vitreous.

### 2.3. MRI Protocol

Structural MRI was performed on a 3T whole-body scanner (GE Signa HDxt, Milwaukee, WI, USA) using an eight-channel phased array head coil. The protocol and acquisition settings have been described previously [31,32]. In brief, the protocol included a three-dimensional T1-weighted fast spoiled gradient echo sequence (FSPGR) for volume measurements and a three-dimensional fluid-attenuated inversion recovery (FLAIR) image for lesion detection. The lesions were segmented automatically on FLAIR using k nearest neighbor classification with tissue type priors (KNN-TTP) [33], and lesion filling was applied on T1 in order to minimize the effect of white matter lesions on atrophy measurements [34]. SIENAX was used to calculate normalized gray and white matter volumes (NGMV and NWMV), as part of the FMRIB Software Library (FSL), http://www.fmrib.ox.ac.uk/fsl (accessed on 8 May 2022).

### 2.4. Statistical Analyses

Statistical analyses were performed using SPSS version 22.0. Data distribution was assessed visually by means of histogram inspection. Because the distribution of VH scores was skewed to the right, all VH scores were log-transformed. For the purpose of assessing disease progression, the study cohort underwent stratification into three groups based on their EDSS scores. Patients were classified as manifesting mild disability, with EDSS scores ranging from 0.0 to 3.5; moderate disability, with scores within the range of 4.0 to 5.5; and severe disability, with scores surpassing 6.0. These thresholds were chosen to reflect the degree of walking impairment [35].

For analyses on the subject level, differences between patients and HCs were tested using linear regression analyses (parametric distribution), the Mann–Whitney U test (non-parametric distribution), or the chi-square test (categorical variables). The mean (±standard deviation, SD) or median (interquartile range (IQR)) are shown. All analyses on eye level (VH and retinal layer thickness, VH and neurodegeneration) were performed using generalized estimating equations with an exchangeable correlation matrix to correct for intra-subject inter-eye correlations. Unless otherwise stated, all analyses were adjusted for age and sex. *p*-Values < 0.05 were considered significant.

## 3. Results

Of the total Amsterdam MS Cohort, 316 patients and 87 HCs had received an OCT scan and were eligible for inclusion in this study. The average age of the cohort was around 50 years, and the female-to-male ratio was approximately 2:1 (Table 1). MRI data were available for the majority of subjects (230 patients and 63 HCs). After quality control, 18 patients were excluded due to poor quality of OCT scans of both eyes. An additional eight patients were excluded due to ocular pathology, leading to a rejection rate of 8.2% (26/316) in total among the patients. Two HCs were excluded from the study, one because of glaucoma and the other because of the poor quality of both macular volume scans. In total, 290 patients with MS and 85 HCs were included in this study. MRI was available for 230 MS patients and 63 HCs. According to the 2010 revised McDonald criteria [36], 200 patients were diagnosed with relapsing–remitting MS (RRMS), 59 with secondary progressive MS (SPMS), and 31 with primary progressive MS (PPMS) (Table 1).

Table 1 shows the characteristics of the cohort. HCs were slightly younger compared to the patients (mean difference 2.2 years, *p* = 0.039). The female-to-male ratio was comparable in both groups (*p* = 0.402). On average, the patients had a relatively long disease duration of 17.9 years, and there was a large variability in the degree of disability (range EDSS score: 0.0–8.5). A relapsing–remitting disease course occurred in 200 patients, while the rest of the patients had a progressive form of MS (SPMS or PPMS, n = 90). The majority of patients (n = 164, 56.6%) had an EDSS score of 0.0–3.5 (mild impairment), 22.1% (n = 64) had an EDSS score of 4.0–5.5 (moderate impairment), and another 21.7% (n = 63) had an EDSS score ≥ 6.0 (severe impairment).

### 3.1. VH and Demographic Characteristics

**Figure 2:**
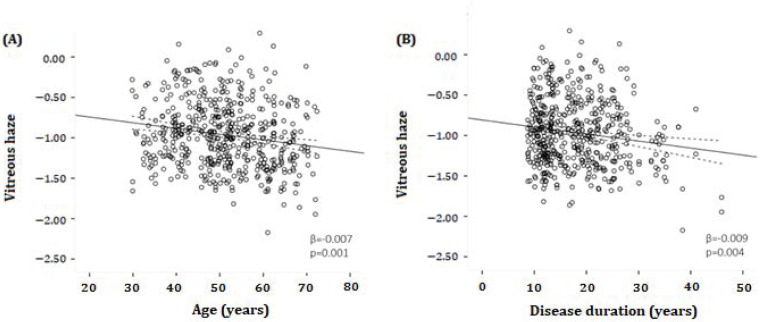
Scatter plots and fitted linear regression lines (with 95% confidence interval) demonstrating the relationship between log-transformed vitreous haze and age (**A**) or disease duration (**B**) in MS patients. (**A**) A significant inverse association was found between vitreous haze and age (β = −0.007, *p* = 0.001). (**B**) A significant inverse association was determined between vitreous haze and disease duration (β = −0.009, *p* = 0.004). β = regression coefficient. All analyses were corrected for age and sex.

Figure 2 shows the scatterplots in which VH (reported as log VH scores) is plotted against age (Figure 2A) and against disease duration (Figure 2B) in MS patients. There was a statistically significant inverse association between VH and both age (β = −0.007, *p* = 0.001) and disease duration (β = −0.009, *p* = 0.004), meaning that a higher age and/or a longer disease duration was associated with lower VH scores in MS patients. There was no significant relationship between VH and age in HCs (β = 0.005, *p* = 0.129) (Table 2).

#### 3.1.1. VH and MS Phenotype

**Figure 3:**
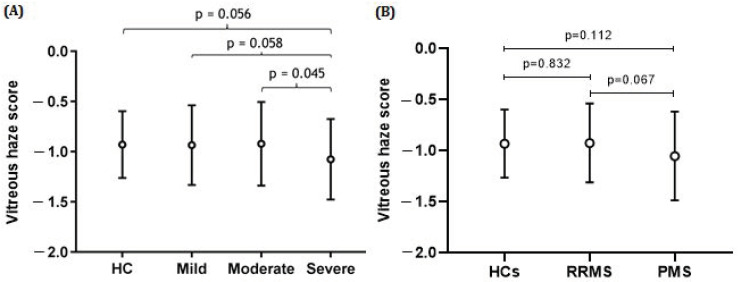
Mean vitreous haze scores (±SD) in MS patients categorized according to disease course and level of physical disability and healthy controls (HCs). The strongest effect is observed for the severe end of the disease spectrum. There was no statistical difference between the controls and persons with MS at the milder end of the disease spectrum. (**A**) The difference in vitreous haze scores between the severe and moderate groups was statistically significant (*p* = 0.045), while the difference between the severe and mild or severe and HC groups was borderline significant (*p* = 0.058; *p* = −0.056). (**B**) The difference in vitreous haze scores between the HC and PMS groups or between the RRMS and PMS groups was not statistically significant; *p* = 0.832 and *p* = 0.067, respectively. All analyses were corrected for age and sex.

On an overall level, there was no significant difference in the amount of VH between MS patients and HCs; the VH score was 9.58% higher in MS patients, *p* = 0.629 (Table 1). No statistical significance difference was found in VH score between RRMS and PMS patients, and RRMS patients and HCs (respectively *p* = 0.067; *p* = 0.832) (Figure 3).

#### 3.1.2. VH and Severity of Disability, Stratified by EDSS

Patients with the highest amount of disability showed the least VH (mean VH score 0.14 (SD ± 0.18)) (Table 1, and Figure 3). Severely affected patients had lower VH than moderately affected patients (−28.86%, *p* = 0.045), while the difference between the severe and mild groups only showed a trend (−23.56%, *p* = 0.058) (see Figure 3).

### 3.2. VH and Retinal Layer Thickness

Compared to HCs, MS patients had a lower pRNFL (mean difference of 10.5 µm, *p* < 0.001) and mGCIPL (mean difference 14.7 of µm, *p* < 0.001) thickness (Table 1). When eyes with a previous history of MSON were excluded, MS patients still had a significantly lower pRNFL (mean difference 6.7 µm, *p* < 0.001) and mGCIPL (mean difference 10.6 µm, *p* < 0.001) thickness compared to HCs. Conversely, the mINL was thicker in patients compared to HCs (mean difference 1.0 µm, *p* = 0.003) (Table 1).

**Figure 4:**
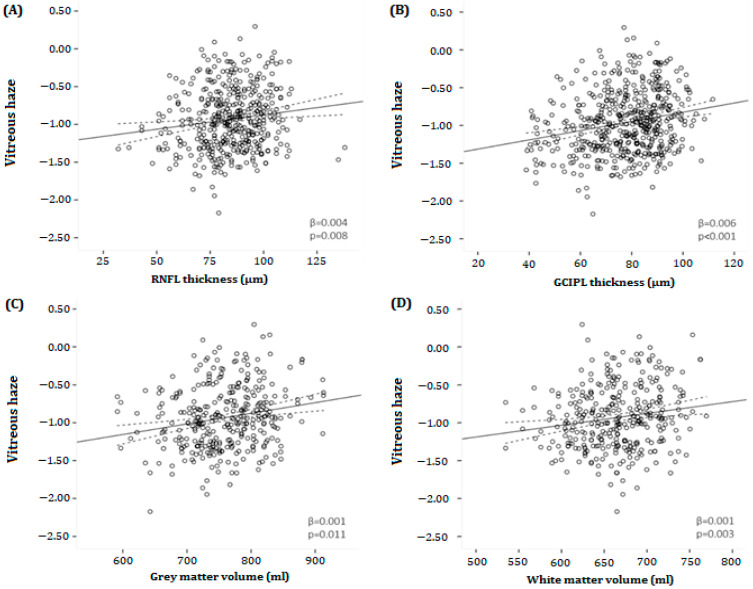
Scatter plots and fitted linear regression lines (and 95% confidence interval) demonstrating the relationship between vitreous haze and (**A**) peripapillary retinal nerve fiber layer thickness (**B**), macular ganglion cell–inner plexiform layer, (**C**) gray, and (**D**) white matter volumes in MS patients. (**A**) A positive association was found between vitreous haze and pRNFL thickness (β = 0.004, *p* = 0.008). (**B**) Likewise, there was a positive relationship between vitreous haze and mGCIPL thickness (β = 0.006, *p* < 0.001). (**C**,**D**) Furthermore, a positive association was found between vitreous haze and both gray as well as white matter volume (β = 0.001, *p* = 0.011; β = 0.001, *p* = 0.003, respectively). mGCIPL = macular ganglion cell–inner plexiform layer; pRNFL = peripapillary retinal; β = regression coefficient. All analyses were corrected for age and sex. nerve fiber layer.

Figure 4A,B shows the relationship between the thickness of the pRNFL and mGCIPL and VH scores in MS patients. There was a significant association between pRNFL thickness and VH in MS patients (β = 0.004, *p* = 0.008) (Table 2). Likewise, in MS patients, there was a significant relationship between mGCIPL thickness and VH (β = 0.006, *p* < 0.001) (Table 2). No significant associations were found between pRNFL or mGCIPL thickness and VH in HCs. When repeating the analyses in the different disability subgroups, the significant association between pRNFL thickness and VH disappeared in both the mild and moderate disability groups (β = 0.003, *p* = 0.154; β = 0.004, *p* = 0.109, respectively), while the relationship between mGCIPL thickness and VH persisted (β = 0.007, *p* = <0.001; β = 0.009, *p* = <0.003, respectively). In the severe disability group, there was a significant association between pRNFL thickness and VH (β = 0.009, *p* = 0.018), as well as between mGCIPL thickness and VH (β = 0.009, *p* = 0.009). Moreover, there was no significant relationship between mINL thickness and VH in HCs, as well as in MS patients, and in all disability groups (*p* = >0.05).

### 3.3. Magnetic Resonance Imaging

Both NGMV (mean difference −4.87%, *p* < 0.001) and NWMV (mean difference −4.74%, *p* < 0.001) were significantly lower in MS patients compared to HC. Figure 4C,D illustrates the relationship between VH and NGMV or VH and NWMV in MS patients. A statistically significant relationship was found between NGMV (β = 0.001, *p* = 0.011), NWMV (β = 0.001, *p* = 0.003), NBV (β = 0.001, *p* = 0.002), and VH, while there was no association between lesion volume and VH (β = −0.058, *p* = 0.416) (Table 2). Again, there was no relationship between NGMV, NWMV, NBV, and VH in HCs.

When repeating the analyses in the different disability subgroups, the significant association between NGMV and VH disappeared in both the mild and moderate disability groups (β = 0.001, *p* = 0.066; β = 0.001, *p* = 0.421, respectively) but remained in the severe disability the group (β = 0.002, *p* = 0.014). The relationship between NWMV or NBV and VH persistently existed in the mild disability group (β = 0.001, *p* = 0.045; β = 0.001, *p* = 0.026, respectively), but disappeared in the moderate (β = 0.002, *p* = 0.127; β = 0.001, *p* = 0.213, respectively) and for NWMV also in the severe disability group (β = 0.002, *p* = 0.095). There was no significant relationship between lesion volume and VH in any of the different disability groups.

## 4. Discussion

In the present study, we investigated the relationship between VH and imaging markers of possible neurodegeneration in patients with MS. A positive relationship was found between the thickness of the inner retinal layers and VH, and it was observed that atrophy of the pRNFL or mGCIPL is associated with lower levels of VH. Furthermore, the relationship between VH and mGCIPL was independent of disability, while the association between VH and pRNFL persisted only in the severe disability group. A positive relationship was also seen between VH and NGMV, NWMV, and NBV, indicating that atrophy of the gray and white matter is associated with lower levels of VH. This was most prominently present in patients with severe disability. In contrast, there was no association between lesion volume and VH.

The interpretation of relations with neurodegenerative markers remains speculative in the absence of in vivo human tracer studies comparable to those done in rodents [20]. The consistently inverse correlation of the VH with markers for neurodegeneration could suggest that instead of acting as a waste bin for neurodegenerative debris, clearance from the vitreous compartment may have increased. Because the glymphatic system is involved in the clearance of neurodegenerative waste products [37,38], such as amyloid beta, the present data, on a group level, may indicate accelerated glymphatic outflow in MS.

There are at least two explanations for the relationship between brain atrophy on MRI and VH. First, this inverse relationship might be due to optic nerve atrophy-related space increase and subsequent acceleration of ocular outflow through the retinal glymphatic system from the eye towards the brain [20]. A clinical observation is that the vitreous body remains quiescent even after severe inner retinal layer atrophy after optic neuropathies. This suggests that neurodegenerative waste products, as known from in vivo human CNS microdialysis studies [39], are rapidly transported away from the extracellular fluid space (ECF). Second, from longitudinal OCT data on the pRNFL and mGCIPL, we know that in almost all persons with MS, there is a degree of pRNFL atrophy in the range of ~7 µm, thought to originate from retrograde trans-synaptic axonal degeneration [40]. The extent of pRNFL atrophy increases further to about 20 µm in persons who also experience MSON [40]. Most of the dynamics of damage happen early in the disease course [41]. Once established, the progression of further atrophy is slow [42]. Therefore, it may be possible that a burnt-out stage of retinal degeneration is reached with longer disease duration and more neurodegeneration, which results in a reduced VH. Furthermore, a build-up of neurodegenerative waste products at the interface between the retina and the vitreous may obliterate transport. Consequently, there would be less accumulation of these neurodegenerative waste products in the vitreous, and VH would decrease. In any of these cases, the likely way of transporting neurodegenerative waste is either through the inner retinal vasculature into the bloodstream or perivascularly along the optic nerve, as described for the ocular glymphatic system [20].

The glymphatic system relies on perivascular astroglia. Their endfeet ensheath penetrating arterioles and venules, creating perivascular spaces through which CSF flows [43]. The exchange of CSF water molecules from the periarterial space to the interstitial space and from the interstitial space to the perivenous space is facilitated by aquaporin-4 channels located on the astrocytic endfeet [44]. It has been proposed that the passage of water molecules through AQP-4 is driven by hydrostatic pressure. CSF solutes subsequently follow by passing either through channels and transporters located on the endfeet or through endfeet clefts [45]. Hydrostatic pressure gradients are a formidable mechanism for explaining glymphatic flow along the optic nerve. A hypothesis is supported by a presumed peripapillary build-up of waste products in cases with impairment of the glymphatic outflow or transient increase of intracranial pressure [46].

**Figure 5:**
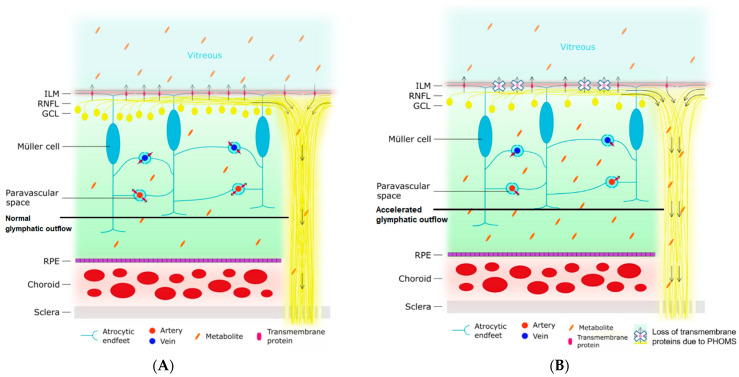
Schematic representation of the proposed ocular glymphatic system and an illustration of how impairment of this system leads to a decline in vitreous haze. The arrows indicate the direction of the flow of the ocular glymphatic system. (**A**) The normal situation is depicted. The endfeet of the Müller cell ensheath retinal arteries and veins, creating perivascular spaces. Likewise, they also form the ILM, which separates the retina from the vitreous. Various transmembrane proteins for the exchange of water and soluble metabolites (e.g., aquaporin-4) are expressed on the astrocytic endfeet. Normally, water enters the retinal tissue from the perivascular space surrounding the retinal arteries. Next, this water exchanges with the interstitial fluid of the retina and picks up metabolites. This fluid, containing the metabolites, then travels to one of three places: (1) the paravascular space surrounding the retinal veins, (2) the vitreous by crossing the ILM, or (3) along the axons of the retinal ganglion cells in the RNFL and the optic nerve. The vitreous haze is caused by metabolites in the vitreous. (**B**) Impaired glymphatic flow in a neurodegenerative state. Neurodegeneration leads to the loss of polarization of transmembrane protein channels at the site of the astrocytic endfeet. In addition, the loss of axons makes the RNFL and optic nerve more permeable. The first process results in a decreased exchange of water and metabolites across the perivascular membranes and the ILM, resulting in less expulsion of metabolites across the ILM into the vitreous and an accumulation of metabolites in the retinal tissue. Secondly, the higher permeability of the RNFL and optic nerve leads to an accelerated outflow along the axons. As a consequence of both processes, there is a decreased amount of metabolites in the vitreous, and thereby less vitreous haze. ILM = inner limiting membrane; RNFL = retinal nerve fiber layer; GCL = ganglion cell layer; RPE = retinal pigment epithelium.

Figure 5 shows a schematic representation of the retinal glymphatic system as demonstrated experimentally, superimposed by the proposed accelerated outflow mechanism through the effects of time and the MS-related atrophied optic nerve. In the retina, homeostasis and metabolism are regulated by Müller cells, a specialized type of retinal glial cell [47]. Similar to the glial cells that form the brain’s glymphatic system, Müller cells express AQP-4 on their membranes [48]. The endfeet of the Müller cells not only ensheath the retinal vasculature and optic nerve but also form the inner limiting membrane separating the retina from the vitreous [49]. Therefore, the retinal glymphatic system exits to two places: the perivenous space (choroid) and the vitreous. In this model, impairment of the retinal glymphatic system would lead to less exchange of neurodegenerative waste products from the retina into the vitreous. In turn, this would lead to a build-up of waste products in the retina and less vitreous haze. However, ocular waste clearance pathways are probably more complex than presently known.

One limitation of our study is that we could not measure the quantitative relationship between neurodegenerative waste products in the vitreous for a correlative analysis with the VH values. In this study, we assumed that the VH is caused by neurodegenerative waste, but we cannot provide definitive evidence in the absence of quantitative protein biomarker data. To investigate experimentally, enucleation will be necessary [50] in order to quantify, for example, neurofilament protein levels in the retina, the adjacent vitreous, or the anterior chamber fluid. For human clinical studies in which an invasive procedure is required on clinical grounds, it has been possible to demonstrate elevated neurofilament protein levels in the vitreous body and anterior chamber fluid [51,52,53]. Such procedures would not be ethical for research purposes in MS.

The strengths of this study are the large cohort of MS patients and the ability of the algorithm to quantify small amounts of VH not readily seen by the naked eye, as illustrated in Figure 1. Another strength is that the conclusions are not based on a single association between VH and a parameter of neurodegeneration, but the results show consistent associations across multimodal imaging parameters (clinical, OCT, and MRI).

## 5. Conclusions

In conclusion, the present study shows an inverse relationship between VH and measures of neurodegeneration in MS. Possible explanations could be the acceleration of the ocular glymphatic outflow from the retina to the brain because the space for drainage increases as the optic nerve volume decreases. Alternatively, one may consider the attainment of a burnt-out stage of neurodegeneration or the clogging up of the retinal vitreous interface. The OCT-based measurement of VH used in this study might, along with other OCT metrics, be of added value in future research investigating the longitudinal dynamics of the retinal glymphatic system in a range of neurodegenerative conditions [37,38,43,44,45,46] and other situations [54,55]. While this study significantly contributes to our understanding of VH and neurodegeneration, future investigations should broaden their scope to examine the impact of treatment, neuropsychiatric, cognition, and blood (bio)markers.

## Figures and Tables

**Table 1:** Characteristics of the study cohort consisting of MS patients and healthy controls.

	MS Patients N = 290	HCs N = 85	*p*-Value
Age (years)	51.5 (±10.1)	49.3 (±8.2)	**0.039**
Sex (Female:Male)	195:95	53:32	0.402
Disease duration (years)	17.9 (±7.0)	N/A	N/A
Time since diagnosis (years)	12.33 (±6.6)	N/A	N/A
Disease course		N/A	N/A
Relapsing–remitting	200 (69.0%)		
Secondary progressive	59 (20.3%)		
Primary progressive	31 (10.7%)		
Medication use at time of visit		N/A	N/A
First-line medication	123 (42.4%)		
Second-line medication	38 (13.1%)		
Unknown	129 (44.5%)		
Impairment using EDSS score		N/A	N/A
Mild (0.0–3.5)	164 (56.6%)		
Moderate (4.0–5.5)	64 (22.1%)		
Severe (≥6.0)	63 (21.7%)		
Optic neuritis		N/A	N/A
No MSON	157 (54.1%)		
Unilateral MSON	81 (27.9%)		
Bilateral MSON	39 (13.4%)		
Unknown	13 (4.5%)		
Relapses in year prior to assessment		N/A	N/A
Yes	34 (11.7%)		
No	256 (88.3%)		
EDSS, median [range]	3.5 [0–8.0]	N/A	N/A
pRNFL thickness (µm)	84.6 (±14.4)	95.1 (±7.9)	**<0.001**
mGCIPL thickness (µm)	77.5 (±14.3)	92.2 (±6.0)	**<0.001**
mINL thickness (µm)	40.4 (±3.3)	39.4 (±2.9)	**0.003**
NGMV (mL)	759.0 (±58.4)	795.5 (±53.3)	**<0.001**
NWMV (mL)	665.2 (±43.8)	696.3 (±33.8)	**<0.001**
Vitreous haze score RR SP PP No MSON Yes MSON EDSS mild EDSS moderate EDSS severe	0.17 (±0.21) 0.18 (0.20) 0.16 (0.22) 0.15 (0.22) 0.17 (0.19) 0.17 (0.25) 0.18 (0.21) 0.19 (0.22) 0.14 (0.18)	0.16 (±0.14)	0.629

Values are shown as mean ± SD unless otherwise stated. Values in bold are significant. MS = multiple sclerosis; HCs = healthy controls; RRMS = relapsing–remitting MS; SPMS = secondary progressive MS; PPMS = primary progressive MS, MSON = MS-associated optic neuritis; MSNON = no history of MS-associated optic neuritis; EDSS = Expanded Disability Status Scale; pRNFL = peripapillary retinal nerve fiber layer; mGCIPL = macular ganglion cell—inner plexiform layer; INL = inner nuclear layer; N/A: not applicable; NGMV = normalized gray matter volume; NWMV = normalized white matter volume. All analyses were corrected for age and sex.

**Table 2:** Relationship between VH and demographic characteristics.

	VH Scores (log) in MS Patients				VH Scores (log) in HC Patients			
Covariable	n	Regression Coefficient	95% Confidence Interval	*p*	n	Regression Coefficient	95% Confidence Interval	*p*
**Age**	520	−0.007	−0.011 to −0.003	**0.001**	161	0.005	−0.001 to 0.012	0.129
**Disease duration**	520	−0.009	−0.015 to −0.003	**0.004**	NA	NA	NA	NA
**Sex:**								
**Female (reference)**	356	1.0	(reference)	---	98	1.0	(reference)	---
**Male**	164	0.014	−0.073 to 0.101	0.748	63	0.090	−0.110 to 0.128	0.881
**Cohort:**								
**HC**	161	1.0	(reference	---	NA	NA	NA	NA
**RRMS**	362	0.008	−0.066 to 0.082	0.833	NA	NA	NA	NA
**SPMS**	102	−0.087	−0.210 to 0.036	0.164	NA	NA	NA	NA
**PPMS**	56	−0.084	−0.229 to 0.060	0.254	NA	NA	NA	NA
**Retinal layer thickness:**								
**pRNFL**	436	0.004	0.001 to 0.006	**0.008**	146	−0.007	−0.015 to 0.000	0.052
**mGCIPL**	370	0.006	0.004 to 0.010	**<0.001**	115	0.007	−0.006 to 0.020	0.294
**mINL**	370	−0.005	−0.019 to 0.009	0.492	115	0.016	−0.007 to 0.039	0.178
**Brain volumes:**								
**Gray matter volume**	339	0.001	0.000 to 0.002	**0.011**	106	−0.001	−0.003 to 0.000	0.143
**White matter volume**	339	0.001	0.000 to 0.002	**0.003**	106	0.000	−0.002 to 0.002	0.845
**Normalized brain volume**	339	0.001	0.0002 to 0.000	**0.002**	174	0.000	−0.002 to 0.001	0.394
**Lesion volume**	339	−0.058	−0.199 to 0.082	0.416	106	NA	NA	NA

Values in bold are significant. MS = multiple sclerosis; HCs = healthy controls; RRMS = relapsing–remitting MS; SPMS = secondary progressive MS; PPMS = primary progressive MS, pRNFL = peripapillary retinal nerve fiber layer; mGCIPL = macular ganglion cell–inner plexiform layer; mINL = macular inner nuclear layer; N/A: not applicable. All analyses were corrected for age and sex.

## Data Availability

The data presented in this study are available upon reasonable request from the corresponding author.
